# Identification of Disulfidptosis‐Associated Hub Genes in Psoriasis via Integrated Transcriptomic and Experimental Validation Approaches

**DOI:** 10.1111/jcmm.70945

**Published:** 2025-11-12

**Authors:** Siyu Liu, Taoyu Chen, Yueyiming Wu, Fangqing Li, Chenyun Wang, Chuanjian Lu, Maojie Wang

**Affiliations:** ^1^ State Key Laboratory of Traditional Chinese Medicine Syndrome The Second Affiliated Hospital of Guangzhou University of Chinese Medicine (Guangdong Provincial Hospital of Chinese Medicine), the Second Clinical Medical College of Guangzhou University of Chinese Medicine Guangzhou China; ^2^ Guangdong‐Hong Kong‐Macau Joint Lab on Chinese Medicine and Immune Disease Research Guangzhou University of Chinese Medicine Guangzhou China; ^3^ State Key Laboratory of Dampness Syndrome of Chinese Medicine The Second Affiliated Hospital of Guangzhou University of Chinese Medicine (Guangdong Provincial Hospital of Chinese Medicine) Guangzhou China; ^4^ Guangdong Provincial Key Laboratory of Clinical Research on Traditional Chinese Medicine Syndrome The Second Affiliated Hospital of Guangzhou University of Chinese Medicine Guangzhou China

**Keywords:** disulfidptosis, glycogen synthase 1, immune infiltration, psoriasis, solute carrier family 3 member 2

## Abstract

Psoriasis is a chronic, immune‐mediated skin disease. While redox imbalance has been implicated in its pathogenesis, the involvement of newly identified cell death pathways remains unclear. Disulfidptosis, a novel form of regulated cell death driven by intracellular disulfide stress, has recently been linked to inflammatory and metabolic diseases, yet its role in psoriasis is unknown. We conducted an integrative analysis combining bulk transcriptomic data (GSE106992 and GSE11239) and single‐cell RNA sequencing data (GSE162183) to identify disulfidptosis‐related genes (DRGs) associated with psoriasis. Differentially expressed genes were intersected with a curated DRG list. Feature selection was performed using LASSO regression and random forest algorithms, followed by WGCNA to identify key modules. Immune infiltration and cell‐type‐specific expression were assessed, and findings were validated using an imiquimod‐induced psoriasis‐like mouse model through Western blotting. To investigate the role of disulfidptosis in psoriasis, we integrated bulk and single‐cell RNA sequencing datasets from psoriatic lesional and non‐lesional skin. Differential expression analysis, WGCNA, and machine learning approaches (LASSO regression and random forest) were employed to identify DRGs. Immune cell infiltration patterns were analyzed, and gene expression was validated in an imiquimod‐induced psoriasis‐like mouse model using Western blotting. Seven DRGs were differentially expressed in psoriatic lesions. Machine learning approaches converged on five candidate genes, with GYS1, SLC3A2, FLNA, FLNB, and TLN1 showing strong discriminatory power (AUC > 0.7). WGCNA identified four hub genes (GYS1, SLC3A2, TLN1, and FLNB) associated with disease‐relevant gene modules. Immune infiltration analysis revealed significant correlations between DRG expression and key immune subsets, including activated CD4^+^ memory T cells and M1 macrophages. Single‐cell RNA‐seq confirmed cell type–specific enrichment of DRGs in epidermal, mesenchymal, and immune cell populations. Protein‐level validation in the murine model further supported transcriptional upregulation of these genes in psoriatic lesions. Our findings suggest that disulfidptosis‐related pathways may contribute to psoriasis pathogenesis through interactions with immune infiltration. The identified hub genes, particularly GYS1 and SLC3A2, represent potential biomarkers and therapeutic targets, offering new insight into the complex molecular landscape of psoriasis.

## 1 Introduction

Psoriasis is a chronic, immune‐mediated, systemic inflammatory disorder that typically manifests as well‐demarcated erythematous plaques covered with silvery scales, often accompanied by pruritus or pain [1]. The global prevalence of psoriasis is estimated at 2%–3%, with variations across different geographic regions and ethnic groups [2]. A hallmark of psoriasis pathogenesis is the hyperproliferation of keratinocytes driven by aberrant immune responses, particularly those involving T cell‐mediated signalling pathways [3]. However, psoriasis is now recognised as a multifactorial disease, with contributions from genetic predisposition, immune dysregulation, environmental stimuli, and neuroendocrine factors. Beyond cutaneous manifestations, patients frequently suffer from a range of systemic comorbidities, including psoriatic arthritis [4], metabolic syndrome [5], mental illness [6], chronic kidney disease [7], cardiovascular disease [8], and malignant tumours [9], all of which significantly impair the quality of life.

In 2023, Liu et al. identified a novel form of programmed cell death, termed disulfidptosis, which is triggered by intracellular accumulation of disulfide stress due to excessive cystine uptake [10]. This process is mediated by the cystine/glutamate antiporter System Xc^−^, which is composed of the subunits SLC7A11(xCT) and SLC3A2 (4F2hc/CD98hc). The activity of this antiporter promotes cystine influx, leading to NADPH depletion and impaired reduction of cystine to cysteine [11]. The resulting disulfide accumulation disrupts cytoskeletal integrity, particularly actin filaments, ultimately leading to cell death. Unlike classical forms of regulated cell death such as apoptosis, ferroptosis, or necroptosis, disulfidptosis operates through a distinct mechanism [10, 11, 12]. Recent research has implicated disulfidptosis‐related genes (DRGs) in the pathogenesis of diverse diseases, including colon cancer [12], ulcerative colitis [13], osteoarthritis [14], non‐alcoholic fatty liver disease [15], and inflammatory bowel disease [16], indicating a broader relevance of disulfide stress in both oncogenic and inflammatory contexts.

Interestingly, increasing attention has been directed toward the role of cysteine metabolism in psoriasis. Disruptions in sulfur amino acid metabolism, particularly elevated homocysteine (Hcy) levels, have been reported in patients with psoriasis and are associated with disease severity and cardiovascular risk [17]. Given that excess intracellular cystine can lead to disulfide bond stress and protein misfolding [18], it is plausible that disulfidptosis may also contribute to the pathological mechanisms underlying psoriasis. Nevertheless, despite the mechanistic overlap between disulfide stress and immune–metabolic imbalance in psoriasis, no prior studies have systematically examined the involvement of disulfidptosis in this disease.

In the present study, we aim to explore the potential role of disulfidptosis in psoriasis by integrating bulk transcriptomic and single‐cell RNA sequencing data from psoriatic lesional and non‐lesional skin. We identified differentially expressed disulfidptosis‐related genes and examined their association with immune cell infiltration. Further, we validated their expression profiles in an imiquimod‐induced mouse model of psoriasis. By uncovering potential regulatory links between disulfidptosis and psoriatic inflammation, this study aims to provide novel insights into the molecular pathogenesis of psoriasis and offer candidate biomarkers or therapeutic targets for future investigation.

## 2 Methods

### 2.1 Identification of Differentially Expressed Genes Between Lesional and Non‐Lesional Skin

Two psoriasis‐associated gene expression datasets (GSE106992 and GSE117239) were obtained from the GEO database (https://www.ncbi.nlm.nih.gov/geo/), comprising 151 lesional and 151 non‐lesional skin samples (68 lesional and 67 non‐lesional in GSE106992; 83 lesional and 84 non‐lesional in GSE117239). Batch effects were corrected using the sva R package, and normalisation was performed with the preprocessCore package. Differential gene expression analysis between lesional and non‐lesional samples was conducted using the limma package. Results were visualised using ggplot2 and pheatmap.

### 2.2 Identification of Disulfidptosis‐Associated Differentially Expressed Genes

A curated list of 24 disulfidptosis‐related genes was compiled based on previous literature [10, 12, 19] (Table S1). Differentially expressed genes (DEGs) were identified from the integrated dataset using the criteria |log_2_FC| > 0.58 and *p* < 0.05. The intersection of DEGs with the disulfidptosis‐related gene list yielded seven target genes. A Venn diagram was used to visualise this overlap.

### 2.3 Functional Enrichment Analysis

Gene Ontology (GO) and Kyoto Encyclopedia of Genes and Genomes (KEGG) enrichment analyses were conducted using the DAVID platform (https://david.ncifcrf.gov/tools.jsp). Enrichment results with *p* ≤ 0.05 were considered statistically significant, allowing interpretation of relevant biological processes and pathways.

### 2.4 Machine Learning Algorithms

To develop a parsimonious gene‐based classifier for psoriasis, we first filtered the transcriptomic matrix to 301 samples based on a predefined hub‐gene list. Least Absolute Shrinkage and Selection Operator (LASSO) logistic regression (glmnet, *α* = 1) with 10‐fold cross‐validation was used to identify genes with non‐zero coefficients at λ_min. In parallel, a Random Forest model (randomForest, 1000 trees) ranked all genes according to the Mean Decrease in Gini index, and the top 10 genes were retained. The intersection of the two gene sets yielded a five‐gene signature (GYS1, SLC3A2, FLNA, FLNB, and TLN1).

A multivariable logistic regression model incorporating these five predictors was then constructed and visualised as a nomogram. Model performance was evaluated in terms of discrimination, calibration, and clinical utility. Discrimination was quantified using receiver operating characteristic (ROC) analysis with area under the curve (AUC). Calibration was assessed using a 1000‐bootstrap calibration plot, and decision curve analysis (DCA, threshold range 0–1) was applied to estimate the net clinical benefit. All performance metrics were reported with bootstrap‐derived 95% confidence intervals.

### 2.5 Weighted Gene Co‐Expression Network Analysis (WGCNA)

The WGCNA package in R was employed to construct a gene co‐expression network. Outlier samples were identified and removed. An appropriate soft‐thresholding power was selected using the pickSoftThreshold function to ensure scale‐free topology. Hierarchical clustering and dynamic tree cutting were applied to define gene modules. Module‐trait relationships were examined based on gene significance (GS) and module membership (MM). Key modules and genes were extracted for subsequent analyses, and eigengene networks were visualised.

### 2.6 Correlation Between Disulfidptosis‐Related Hub Genes and Immune Cell Infiltration

Immune cell fractions were estimated using CIBERSORT (v1.06) [20]. The normalised expression matrix (TPM) was log_2_‐transformed using voom. The LM22 leukocyte signature matrix, representing 22 immune cell subsets, was interrogated with 1000 permutations and quantile normalisation enabled (QN = TRUE). Only samples with a CIBERSORT global *p* value < 0.05 were retained for downstream analysis. The inferred relative proportions of immune cells were used for: (i) between‐group comparisons using the Wilcoxon rank‐sum test with Benjamini–Hochberg false discovery rate (FDR) correction, and (ii) Spearman correlation analyses between immune cell fractions and hub‐gene expression levels, with FDR adjustment across all 22 immune subsets (Table S1).

### 2.7 Single‐Cell Sequencing Analysis

Single‐cell RNA‐sequencing (scRNA‐seq) data were filtered to retain cells expressing 300–6000 genes, containing < 15% mitochondrial transcripts, and < 20,000 unique molecular identifiers (UMIs). Doublets were identified and removed using Scrublet (threshold = 0.25). Filtered datasets were integrated with Harmony (v0.1.0) using donor ID as the batch variable, followed by principal component analysis (PCA) and graph‐based clustering (Seurat v5, resolution = 0.6). Cluster annotation was based on canonical lineage markers cross‐referenced with the top differentially expressed genes within each cluster. Five major populations were defined: Endothelial (Endo) – CDH5, PECAM1, CD34, Epidermal (EpD) – KRT10, KRT14, KRT1, Immune (IM) – PTPRC, ITGAM, CD3E, Mesenchymal (Mes) – PDGFRB, LUM, COL1A1, Neural‐crest‐like (SchM) – SOX10, MLANA, S100B.

### 2.8 Animal Experiments

Male C57BL/6 mice (20 ± 2 g) were provided by the Laboratory Animal Center of Southern Medical University (Guangzhou, China). Mice were housed under standard specific‐pathogen‐free (SPF) conditions (22°C ± 2°C, 45%–55% humidity, 12 h light/dark cycle) with free access to food and water. All experimental protocols were approved by the Animal Experimental Ethics Committee of Guangdong Provincial Hospital of Chinese Medicine (Animal ethics number ID:2024128).

### 2.9 Imiquimod‐Induced Psoriasis‐Like Mouse Model

To induce psoriasis‐like inflammation, 62.5 mg of 5% imiquimod cream was topically applied daily to a shaved area of dorsal skin (3 × 2.5 cm) for seven consecutive days. On day 7, disease severity was evaluated using the Psoriasis Area and Severity Index (PASI), scoring erythema, scaling, and thickness on a 0–4 scale (0 = none; 4 = very marked).

### 2.10 Western Blotting

Tissue lysates were prepared in NP‐40 buffer, sonicated, centrifuged, and quantified. Proteins were denatured at 95°C for 10 min and separated via SDS‐PAGE. After transfer to PVDF membranes, blots were incubated with primary and HRP‐conjugated secondary antibodies. For non‐reducing conditions, aliquots were processed at 70°C for 10 min without reducing agents.

### 2.11 RNA Extraction and Quantitative Real‐Time PCR

Total RNA was extracted using TRIzol reagent (Life Technologies, CA, USA) and reverse‐transcribed into cDNA using the iScript cDNA Synthesis Kit (Takara, Liaoning, China). qPCR was conducted with SYBR Premix Ex Taq (Takara) on an ABI 7500 system (Thermo Fisher Scientific, MA, USA). Primer sequences are listed in Table S2.

### 2.12 Histological Analysis

Skin samples were fixed in 4% paraformaldehyde, embedded in paraffin, and sectioned at 5 μm thickness. Haematoxylin and eosin (H&E) staining was performed to assess epidermal morphology and inflammation. Epidermal thickness was quantified using Fiji/ImageJ by measuring the distance from the basal to the stratum corneum at five randomly selected, vertically oriented sites per section. The mean value for each sample was calculated and used for statistical analysis.

### 2.13 Statistical Analysis

Statistical analyses were performed using R (version 4.3.2), with *p* < 0.05 considered statistically significant.

## 3 Results

### 3.1 Identification of Differentially Expressed Genes and Pathway Enrichment in Psoriatic Lesional Skin

**FIGURE 1:**
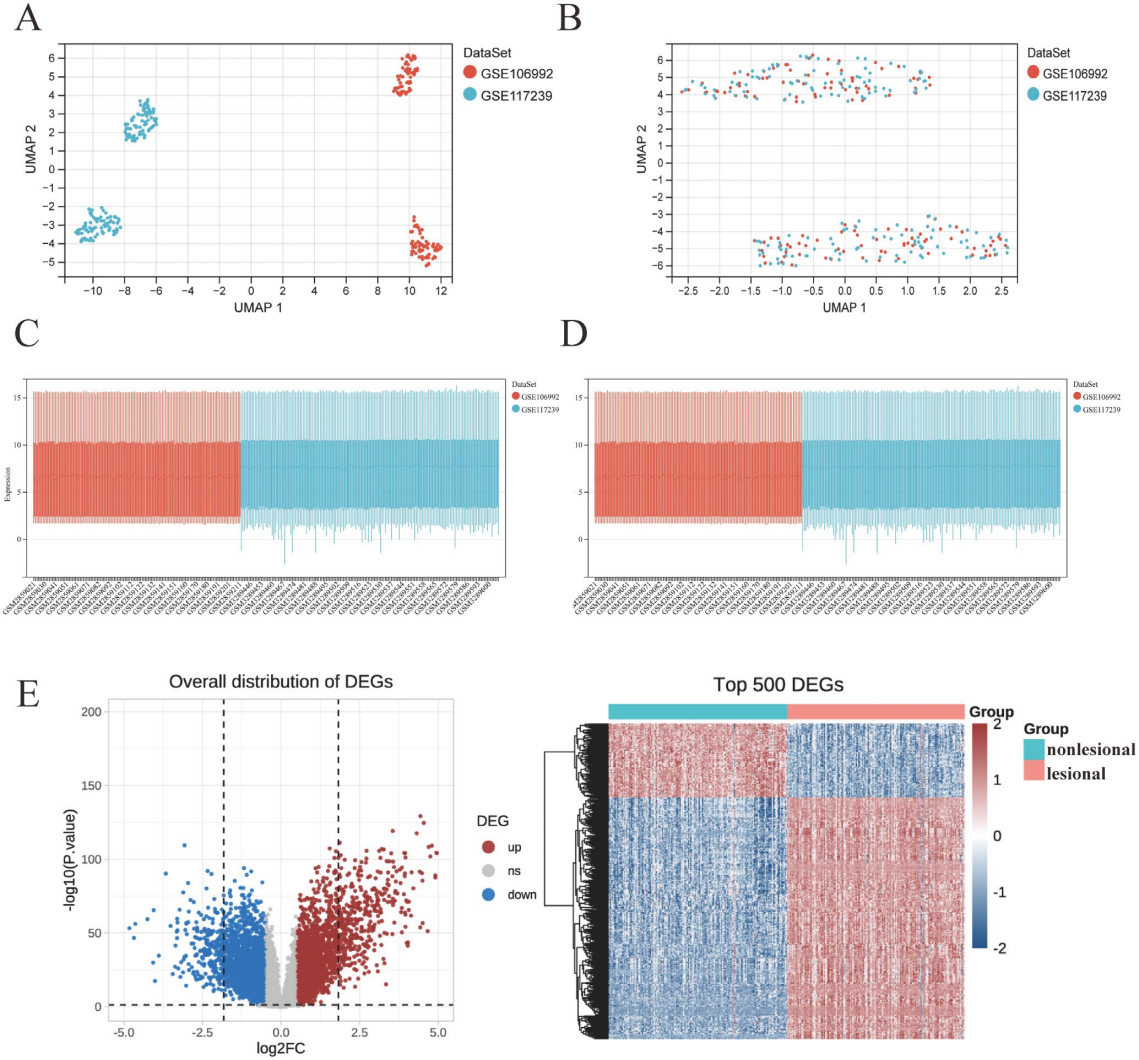
Global transcriptomic landscape of psoriatic skin. (A, B) UMAP plots showing sample clustering before (A) and after (B) batch correction of gene expression data. (C, D) Bar plots of the number of differentially expressed genes (DEGs) identified before (C) and after (D) normalisation. (E) Volcano plot of DEGs with adjusted *p*‐value (adj. *p* val) thresholds; grey dots represent non‐significant genes (adj. *p* val > 0.05). (F) Heatmap displaying representative DEGs between lesional and non‐lesional skin from psoriasis patients.

We obtained two microarray datasets (GSE106992 and GSE117239) from the GEO database, both containing gene expression profiles from lesional and non‐lesional skin biopsy samples of patients with psoriasis, totaling 302 samples (151 pairs). After batch effect correction (Figure 1A–D), the datasets were integrated for subsequent analysis. Differential expression analysis was conducted using a threshold of |log_2_ fold change| > 0.58 and adjusted *p*‐value < 0.05, identifying 2546 upregulated and 2843 downregulated genes in psoriatic lesional tissues compared to non‐lesional controls (Figure 1E,F). These differentially expressed genes (DEGs) were subjected to Gene Ontology (GO) enrichment analysis (Figure S1A–C), revealing significant associations with signal transduction, apoptotic processes, and identical protein binding. KEGG pathway analysis further implicated the TNF and PPAR signalling pathways in psoriasis pathogenesis (Figure S1D).

### 3.2 Identification of Disulfidptosis‐Related Genes in Psoriatic Lesions

**FIGURE 2:**
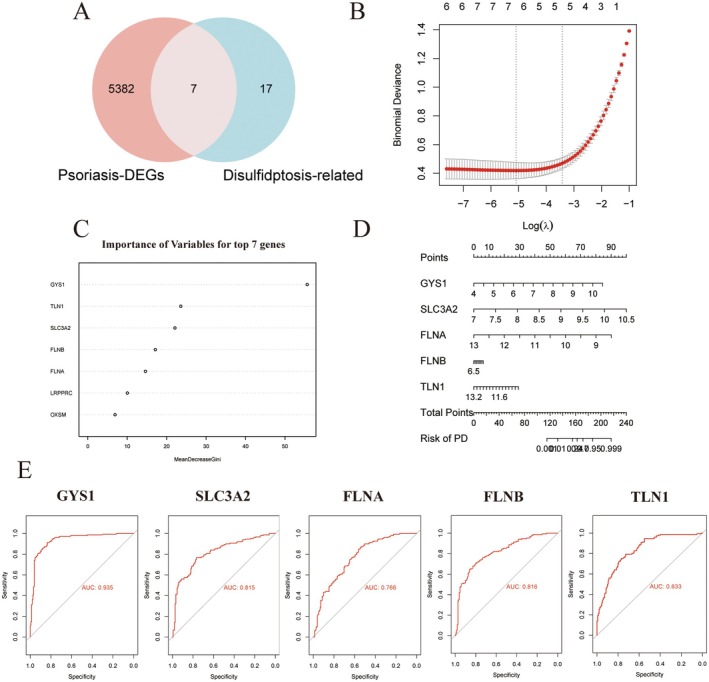
Identification of Disulfidptosis‐related genes (DRGs) in psoriasis. (A) Venn diagram showing the overlap between psoriasis‐related DEGs and known disulfidptosis‐related genes, identifying seven intersecting genes. (B) LASSO regression analysis for gene selection. (C) Random forest classifier identifying 10 candidate genes. (D) Nomogram integrating five key genes for predicting psoriasis risk. (E) ROC curves illustrating the diagnostic performance of the five selected genes.

To investigate whether disulfidptosis occurs in psoriatic lesional tissues, we compared a set of disulfidptosis‐related genes (DRGs) [10, 12, 19] (Table S1) with the psoriasis‐associated DEGs, identifying seven overlapping genes. We then applied LASSO regression and random forest algorithms to prioritise DRGs based on their discriminative power between lesional and non‐lesional samples of psoriasis. These analyses identified seven (Figure S2A and Figure 2B) and ten candidate genes (Figure 2C), respectively, with five genes, glycogen synthase 1 (GYS1), solute carrier family 3 member 2 (SLC3A2), Filamin A (FLNA), FLNB, and Talin 1 (TLN1), shared between both methods. As illustrated by the nomogram in Figure 2D, this five‐gene signature demonstrates strong potential for distinguishing lesional from non‐lesional tissue in psoriasis. Receiver operating characteristic (ROC) curves and corresponding area under the curve (AUC) values for each gene are shown in Figure 2E. All five genes exhibited AUCs greater than 0.7, with GYS1 achieving the highest (AUC = 0.935), followed by TLN1 (AUC = 0.833). These findings underscore the differential expression of DRGs in psoriatic lesions and highlight their potential diagnostic value.

### 3.3 WGCNA Identifies Core DRGs Potentially Involved in Psoriasis Pathogenesis

**FIGURE 3:**
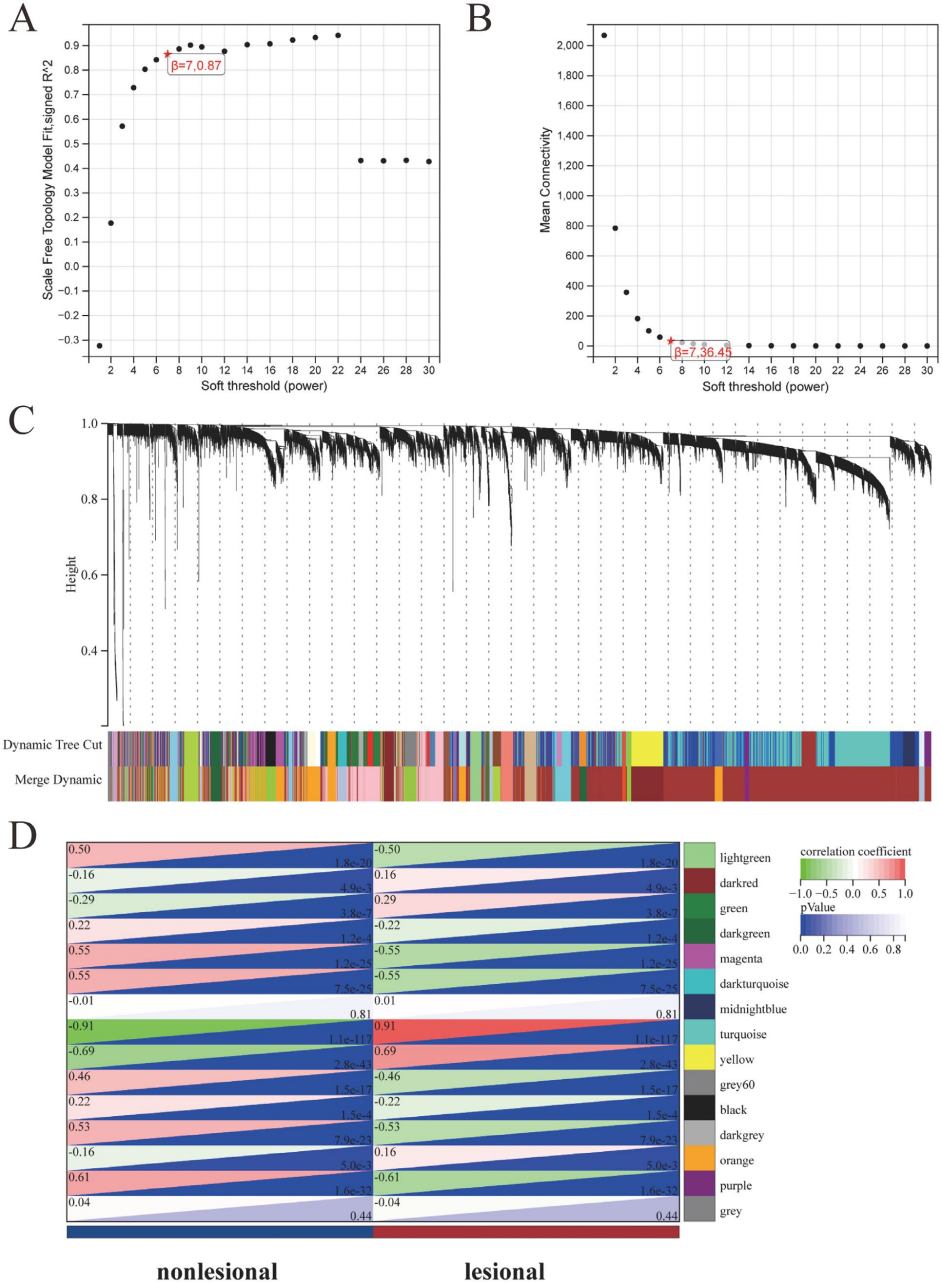
Weighted Gene Co‐expression Network Analysis (WGCNA). (A, B) Scale‐free topology fit index and mean connectivity for a range of soft threshold powers. (C) Gene dendrogram showing clustering into modules, each represented by a distinct colour. (D) Heatmap displaying correlations between module eigengenes and psoriasis lesion status; red indicates positive, green negative correlation.

To further explore gene expression patterns in psoriatic lesional versus non‐lesional skin, we performed Weighted Gene Co‐expression Network Analysis (WGCNA) using the merged datasets GSE106992 and GSE117239. Based on the scale‐free topology criterion, a soft‐thresholding power of seven was selected using the sft$powerEstimate function, yielding a scale‐free topology fit index of R^2^ = 0.87 with satisfactory mean connectivity (Figure 3A,B). A co‐expression network was then constructed using the one‐step method in the WGCNA R package, with parameters set as follows: soft‐threshold power = 7, minimum module size = 50, and deepSplit = 2 (Figure 3C and Figure S2C). The resulting modules showed strong independence, as confirmed by the topological overlap matrix and corresponding dendrogram heatmap (Figure S2C).

Module‐trait correlation analysis identified the turquoise module as most strongly associated with psoriatic lesional skin (*r* = 0.91, *p* < 1 × 10^−100^; Figure 3D and Figure S2D), suggesting its potential relevance to disease pathogenesis. To pinpoint key regulatory genes, we intersected three gene sets: psoriasis‐related DEGs, genes from the turquoise module, and disulfidptosis‐related genes. Venn diagram analysis revealed four overlapping genes, SLC3A2, GYS1, FLNA, and FLNB, which may serve as core candidates linking disulfidptosis to the molecular mechanisms of psoriasis (Figure S2B).

### 3.4 Disulfidptosis‐Related Genes Are Associated With Immune Cell Infiltration in Psoriatic Skin

**FIGURE 4:**
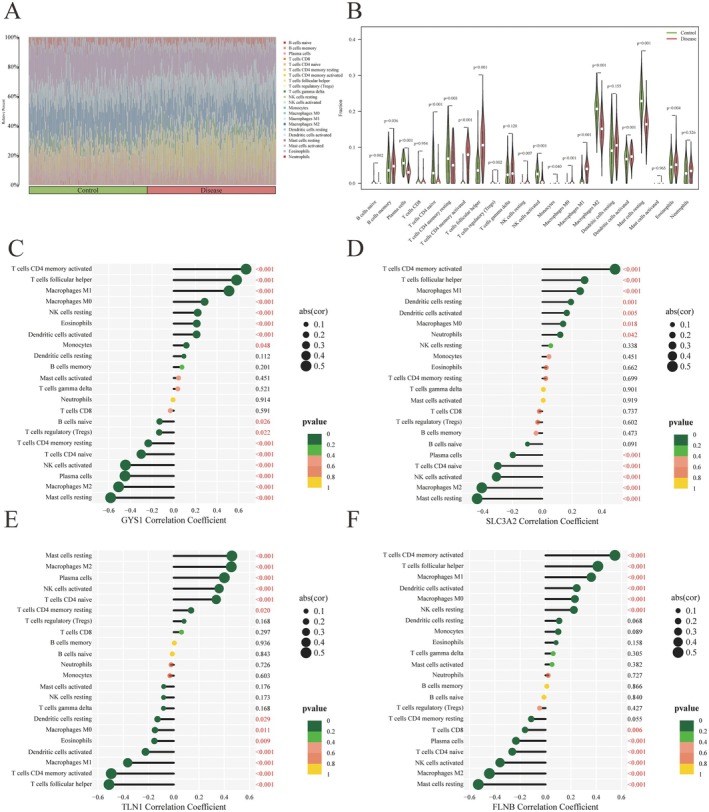
Correlation between hub genes and immune cell infiltration. (A, B) Heatmap (A) and bar plot (B) showing differences in immune cell infiltration between psoriatic lesional and non‐lesional skin. (C–F) Spearman correlation analyses between immune cell populations and expression of hub genes: (C) GYS1, (D) SLC3A2, (E) TLN1, and (F) FLNB.

To characterise the immune landscape of psoriatic lesional skin, we conducted immune cell infiltration analysis comparing lesional and non‐lesional samples. A total of 23 immune cell subsets were assessed. As shown in the bar and violin plots, six immune cell types, including activated CD4^+^ memory T cells and T follicular helper cells, were significantly elevated in lesional skin, whereas eight subsets, such as resting mast cells and naïve B cells, exhibited markedly reduced infiltration (Figure 4A,B and Figure S3A).

We further examined the association between the expression of four hub genes (GYS1, SLC3A2, TLN1, and FLNB) and immune cell infiltration. GYS1 expression was significantly positively correlated with eight immune cell subsets, most notably with activated CD4^+^ memory T cells, T follicular helper cells, and M1 macrophages. On the other hand, it showed significant negative correlations with six cell types, particularly resting dendritic cells and monocytes (Figure 4C). SLC3A2 expression exhibited strong positive correlations with seven immune cell types, including activated CD4^+^ memory T cells and T follicular helper cells, while it was negatively correlated with five subsets, especially resting dendritic cells and resting NK cells (Figure 4D). TLN1 expression was positively correlated with six immune cell types, most notably resting mast cells and M2 macrophages. It also showed strong negative associations with activated CD4^+^ memory T cells and T follicular helper cells (Figure 4E). FLNB was significantly positively associated with six immune cell types, particularly activated CD4^+^ memory T cells, T follicular helper cells, and M1 macrophages, while its expression was negatively correlated with six subsets, especially activated and resting dendritic cells (Figure 4F).

Notably, GYS1, SLC3A2, and FLNB were strongly and positively correlated with the infiltration of activated CD4^+^ memory T cells, suggesting their involvement in psoriatic immune activation. In contrast, TLN1 expression was positively associated with resting mast cell abundance, indicating a potential role in local immune modulation. These findings underscore the potential involvement of disulfidptosis in regulating immune cell infiltration within psoriatic lesions.

### 3.5 Disulfidptosis‐Related Hub Genes Are Upregulated in Psoriatic Lesions Compared to Healthy Skin

**FIGURE 5:**
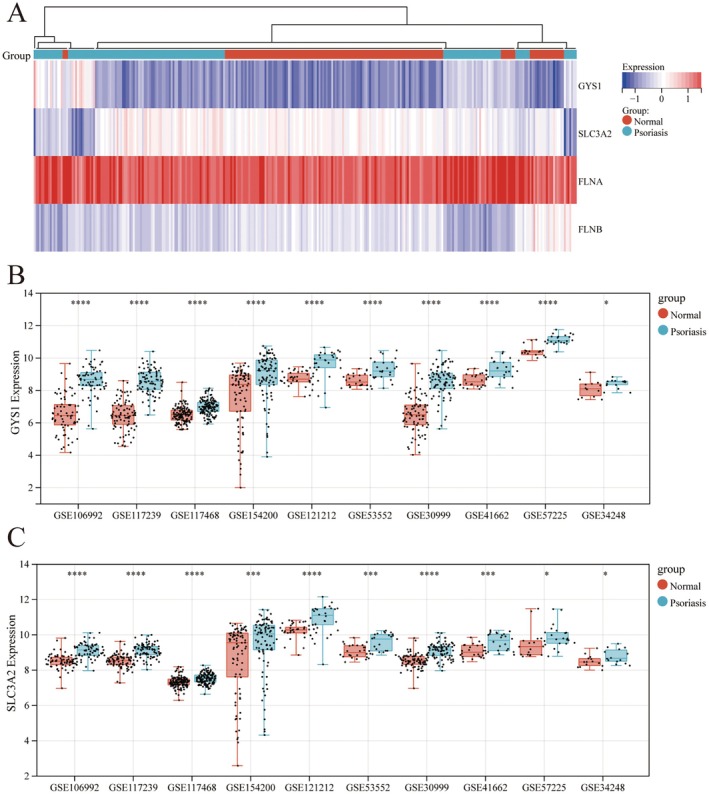
Hub gene expression profiles in psoriasis datasets. (A) Heatmap of hub gene expression (GYS1, SLC3A2, TLN1, FLNB) in GSE106992 and GSE117239 datasets. (B, C) Expression levels of (B) GYS1 and (C) SLC3A2 across multiple GEO psoriasis datasets.

Based on the analysis of the GSE106992 and GSE117239 datasets, we confirmed that the hub genes (GYS1, SLC3A2, TLN1, and FLNB) were significantly upregulated in psoriatic lesional tissue compared to non‐lesional skin (Figure 5A). However, their expression patterns relative to healthy skin remained unclear. To address this, we incorporated additional GEO datasets containing both healthy skin samples and psoriatic lesional samples for further analysis. The results showed that the expression levels of GYS1 (Figure 5B), SLC3A2 (Figure 5C), TLN1 (Figure S3A), and FLNB (Figure S3B) were consistently elevated in psoriatic lesions compared to healthy skin. Notably, GYS1 and SLC3A2 demonstrated statistically significant upregulation in all datasets analyzed (Figure 5B,C). These findings further underscore the potential importance of these four disulfidptosis‐related hub genes in the pathogenesis of psoriasis.

### 3.6 Single‐Cell Transcriptomic Profiling Reveals Cell Type–Specific Expression of Disulfidptosis‐Related Hub Genes in Psoriatic Lesions

**FIGURE 6:**
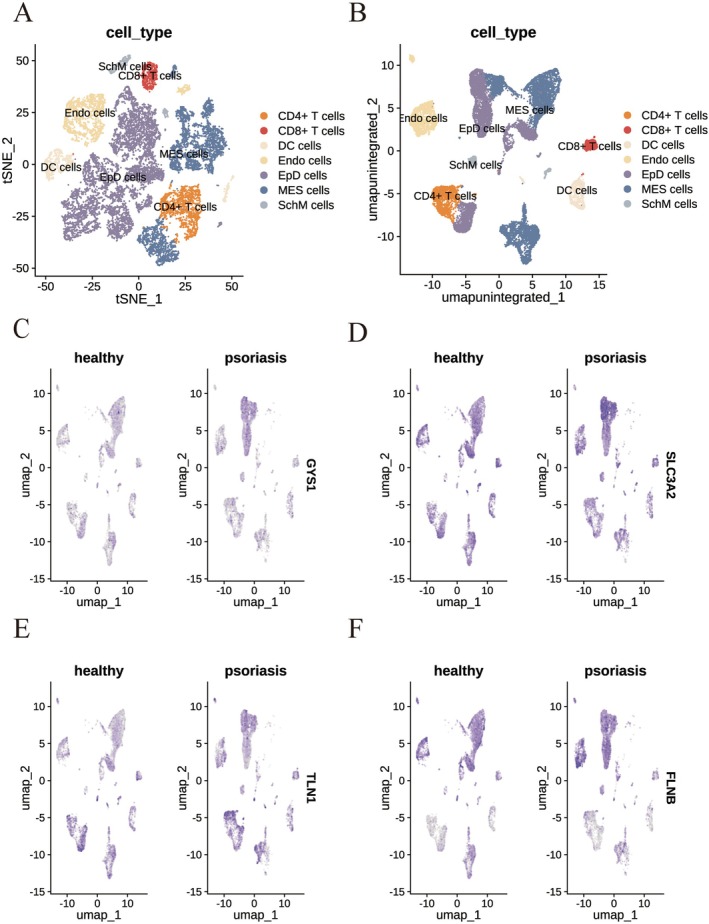
Single‐cell validation of disulfidptosis‐related hub genes. (A) t‐SNE and (B) UMAP plots visualising single‐cell transcriptomic data (GSE162183) for overall cellular distribution in psoriatic skin. (C–E) UMAP plots showing the expression of DRGs in lesional vs. control samples: (C) GYS1, (D) SLC3A2, (E) TLN1, (F) FLNB, confirming their expression in specific cell subsets of psoriatic skin.

In our immune cell infiltration analysis, we observed significant correlations between the four disulfidptosis‐related hub genes and various immune cell populations. To further investigate the cellular basis of these associations, we analysed single‐cell transcriptomic data from psoriatic lesional tissue (GSE162183), using healthy skin samples as controls. The goal was to determine the specific immune cell subsets in which these hub genes are expressed. We conducted single‐cell analysis using the Seurat package, applied t‐SNE and UMAP algorithms for clustering, and annotated cell types based on the HumanPrimaryCellAtlasData reference using the SingleR package. Cells were classified into seven major categories: CD4^+^ T cells, CD8^+^ T cells, dendritic cells (DCs), endothelial cells (Endo), epidermal cells (EpD), mesenchymal cells (MES), and Schwann/mesenchymal‐like cells (SchM) (Figure 6A and 6B).

We then visualised the expression patterns of the four hub genes across these cell subsets on the UMAP plot. GYS1 was predominantly expressed in MES and EpD cells, with notably higher expression in EpD cells from psoriatic lesions compared to healthy skins (Figure 6C and Figure S4A). SLC3A2 showed moderate expression across multiple cell types but was particularly enriched in psoriatic EpD cells (Figure 6C and Figure S4B). TLN1 was mainly expressed in MES, EpD, and CD4^+^ T cells; notably, CD4^+^ T cells from psoriatic skin exhibited elevated TLN1 expression relative to healthy skins (Figure 6E and Figure S4C). FLNB was primarily expressed in Endo, MES, and EpD cells, but its expression levels did not differ significantly between psoriatic and healthy skins (Figure 6A,F and Figure S4D). These results reveal distinct expression patterns of disulfidptosis‐related hub genes across different cell subsets and suggest that disulfidptosis may exert cell type‐specific effects in the pathogenesis of psoriasis.

### 3.7 Validation of Disulfidptosis‐Related Hub Genes in an Imiquimod‐Induced Psoriasis‐Like Mouse Model

**FIGURE 7:**
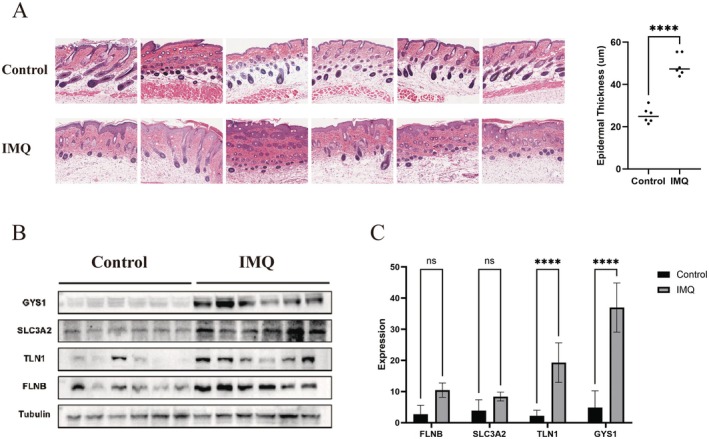
Validation of disulfidptosis‐related hub genes in psoriatic mouse model. (A) H&E staining of skin tissue sections from different groups. Epidermal thickness in lesions of mice was measured by the Fiji ImageJ software and depicted as mean ± SD of *n* = 6 per group, ****p < 0.0001. (B) Western blot of disulfidptosis‐related hub genes expression in skin samples from different groups. Control, *n* = 6 mice; IMQ, *n* = 6 mice. (C) Quantitative Real‐Time PCR of disulfidptosis‐related hub genes expression in skin samples from different groups. Control, *n* = 6 mice; IMQ, *n* = 6 mice. Data are shown as mean ± SD; not significant (ns), *p* > 0.05; ****p < 0.0001.

Finally, we validated the expression levels of the four disulfidptosis‐related hub genes in an established imiquimod (IMQ)‐induced murine model of psoriasis‐like skin inflammation. Haematoxylin and eosin staining of skin tissue sections revealed classical psoriatic histopathological features, including epidermal hyperplasia, abnormal keratinization, inflammatory cell infiltration, and increased vascularization, confirming successful model induction (Figure 7A). Subsequently, protein was extracted from the skin tissues of IMQ‐treated and control mice, and Western blot analysis was performed to assess the protein expression of GYS1, SLC3A2, TLN1, and FLNB. Consistent with the transcriptomic findings, all four proteins were upregulated in IMQ‐induced psoriatic skin (Figure 7B). Among them, GYS1 and FLNB showed the most prominent increases, with statistically significant differences compared to controls (Figure 7C). These results provide further evidence at the protein‐level that disulfidptosis‐related hub genes are upregulated in psoriatic‐like pathological tissue.

## 4 Discussion

Psoriasis is a chronic, immune‐mediated skin disorder characterised by hyperproliferation of keratinocytes, infiltration of immune cells, and aberrant angiogenesis. While its immunological basis is well established, recent studies have also highlighted the contribution of metabolic stress and redox imbalance to its pathogenesis. Disulfidptosis, a novel form of regulated cell death driven by the accumulation of intracellular disulfide molecules such as cystine, has been implicated in various malignancies [10, 12]. However, its relevance to non‐cancerous inflammatory diseases, including psoriasis, remains largely unexplored.

In this study, we systematically integrated multiple transcriptomic datasets from psoriatic lesional and non‐lesional tissues to investigate the potential involvement of disulfidptosis in psoriasis. Using differential expression analysis, WGCNA, and machine learning approaches (LASSO and random forest), we identified four core disulfidptosis‐related genes, GYS1, SLC3A2, TLN1, and FLNB, that were consistently upregulated in psoriatic lesions. These genes demonstrated strong discriminatory power between psoriatic lesional and non‐lesional skin and were further validated across independent datasets and an imiquimod‐induced psoriasis‐like mouse model.

Among the hub genes, GYS1, which encodes glycogen synthase 1, plays a central role in glycogen metabolism and cellular redox homeostasis. Previous studies have shown that its overexpression promotes disulfidptosis in tumour cells by altering NADPH levels and inducing oxidative stress [20]. Its strong upregulation in psoriatic epidermal and mesenchymal cells, together with its positive correlation with proinflammatory immune subsets such as activated CD4^+^ memory T cells and M1 macrophages, suggests that GYS1 may exacerbate local inflammation through metabolic reprogramming and redox imbalance. SLC3A2, another identified gene, is a transmembrane protein involved in amino acid transport and integrin signalling. It has been shown to promote tumour growth and immune cell infiltration in multiple cancers [21, 22]. In psoriasis, SLC3A2 was enriched in epidermal cells and exhibited strong positive correlations with T follicular helper cells and CD4^+^ memory T cells, two immune subsets central to psoriatic inflammation. These findings raise the possibility that SLC3A2 may facilitate inflammatory cell recruitment and activation in the psoriatic microenvironment. TLN1 and FLNB, both cytoskeletal proteins involved in actin filament organisation and cell adhesion, were also found to be upregulated in psoriatic lesional skin. TLN1 has been associated with enhanced cellular migration and integrin activation in cancers such as AML and prostate cancer [23, 24]. FLNB mutations are known to disrupt cytoskeletal dynamics and are implicated in skeletal dysplasia syndromes [25]. In the context of psoriasis, their elevated expression may reflect increased cytoskeletal stress and aberrant adhesion signalling in keratinocytes, contributing to hyperplasia and altered differentiation [26].

Beyond gene‐level expression, immune infiltration analysis revealed that GYS1, SLC3A2, and FLNB were significantly correlated with inflammatory immune cells, particularly memory CD4^+^ T cells and M1 macrophages, suggesting a potential link between disulfidptosis and immune activation. Notably, TLN1 showed an inverse pattern, correlating positively with resting mast cells, implying a potential immunoregulatory role. Single‐cell RNA sequencing provided further insights into the cellular specificity of these genes. GYS1 and SLC3A2 were highly expressed in epidermal cells from psoriatic lesions, aligning with previous reports of keratinocyte metabolic reprogramming in psoriasis [27]. TLN1 showed elevated expression in both mesenchymal and CD4^+^ T cells, underscoring its multifaceted role in epithelial‐immune crosstalk. Interestingly, FLNB was broadly expressed but did not show significant changes between healthy and lesional tissues at the single‐cell level, suggesting it may contribute to structural integrity rather than inflammation per se. Finally, Western blot analyses in an imiquimod‐induced psoriasis mouse model confirmed the upregulation of all four hub proteins, particularly GYS1 and FLNB, providing translational support for our transcriptomic findings.

Despite the strength of our integrative approach, this study has several limitations. Most notably, our analysis remains correlative, and the mechanistic link between disulfidptosis and psoriatic inflammation requires further experimental validation. Future studies should aim to dissect how modulation of disulfidptosis‐related genes affects keratinocyte survival, immune cell activation, and redox homeostasis using in vitro and in vivo functional models.

## 5 Conclusion

In summary, our study identifies a set of disulfidptosis‐related gene signatures that are significantly associated with psoriasis. By integrating multiple transcriptomic datasets and experimental validation, we revealed that four genes (GYS1, SLC3A2, TLN1, and FLNB) are consistently upregulated in psoriatic lesions and exhibit strong correlations with immune activation and metabolic remodelling. These findings indicate an association between disulfidptosis‐related molecular features and psoriatic inflammation, although causal relationships remain to be determined through further experimental studies. With additional functional validation, these genes may serve as potential biomarkers and offer new insights into the metabolic–immune interactions underlying psoriasis.

## Author Contributions

**Siyu Liu:** data curation (equal), writing – original draft (equal). **Taoyu Chen:** visualization (equal), writing – original draft (supporting). **Yueyiming Wu:** investigation (equal), visualization (equal). **Fangqing Li:** data curation (equal), resources (equal). **Chenyun Wang:** investigation (equal), software (equal). **Chuanjian Lu:** funding acquisition (equal), supervision (equal). **Maojie Wang:** conceptualization (equal), writing – original draft (equal).

## Conflicts of Interest

The authors declare no conflicts of interest.

## Supporting information


**Figure S1:** Functional enrichment of DEGs in psoriasis.
**Figure S2:** Identification of disulfidptosis‐related gene modules in psoriasis.
**Figure S3:** Expression validation of TLN1 and FLNB in psoriasis datasets.
**Figure S4:** Disulfidptosis‐related hub genes expression in different cell subsets.

**Table S1:** disulfidptosis‐related genes.

**Table S2:** Disulfidptosis‐related genes in psoriatic lesions and their primer sequence.

## Data Availability

All data are available in the main text or the Supporting Information.
