# Supplementary material for: Identification of Disulfidptosis‐Associated Hub Genes in Psoriasis via Integrated Transcriptomic and Experimental Validation Approaches
Source: J Cell Mol Med. 2025 Nov 12;29(21):e70945. doi: 10.1111/jcmm.70945 (PMC12611608; doi:10.1111/jcmm.70945)
Supplement: Supplementary file 1 — Figure S1: Functional enrichment of DEGs in psoriasis. Figure S2: Identification of disulfidptosis‐related gene modules in psoriasis. Figure S3: Expression validation of TLN1 and FLNB in psoriasis datasets. Figure S4: Disulfidptosis‐related hub genes expression in different cell subsets. [file JCMM-29-e70945-s003.zip › Figure S1-S4.docx]

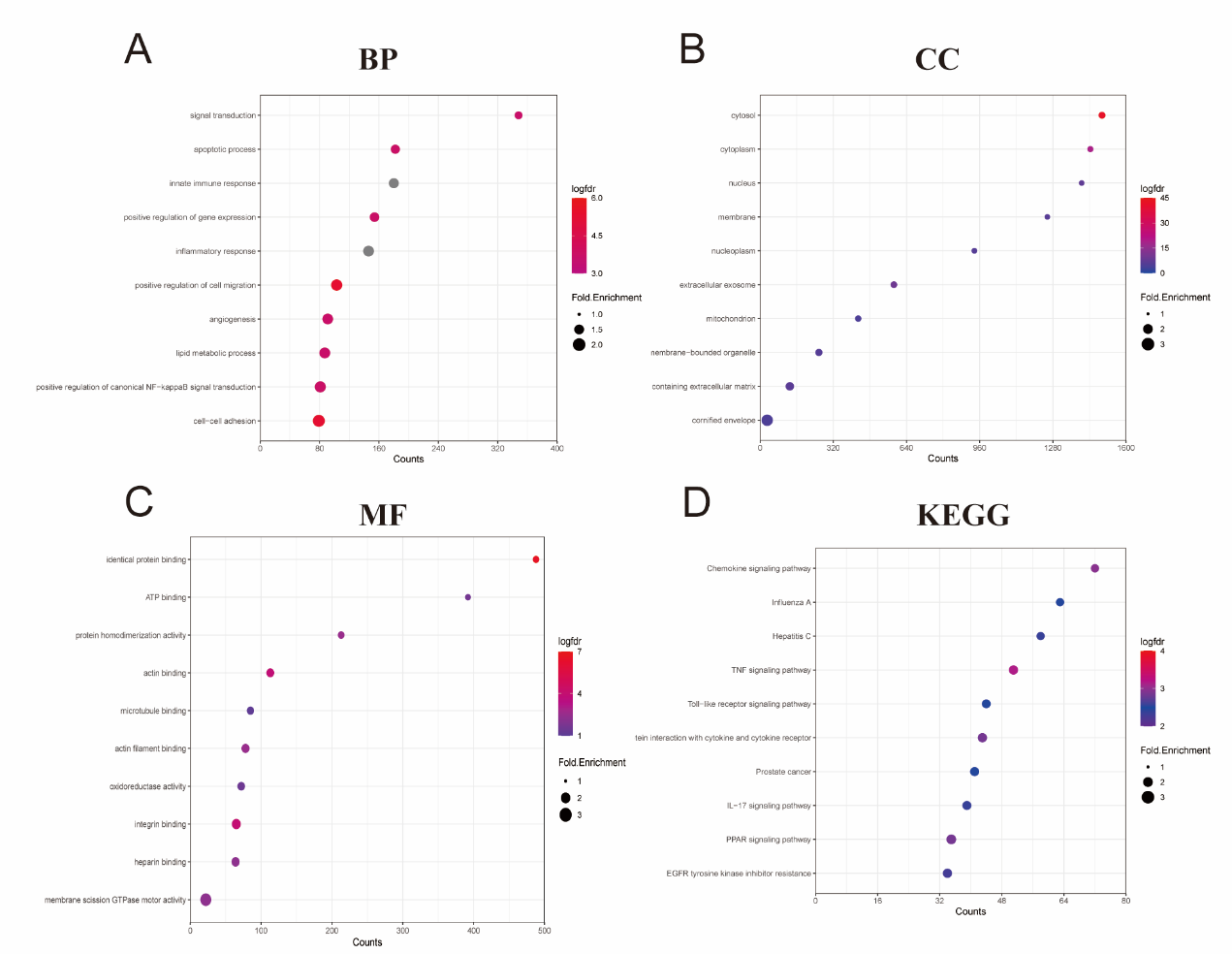


**Figure S1. Functional enrichment of DEGs in psoriasis.**

(A) Biological processes (BP) enriched for immune activation and cell migration.

(B) Cellular components (CC) including cytosol, exosome, and nucleus.

(C) Molecular functions (MF) related to ATP and actin binding, and cytokine activity.

(D) KEGG pathways enriched in IL-17, TNF signaling, and viral infection responses.

**
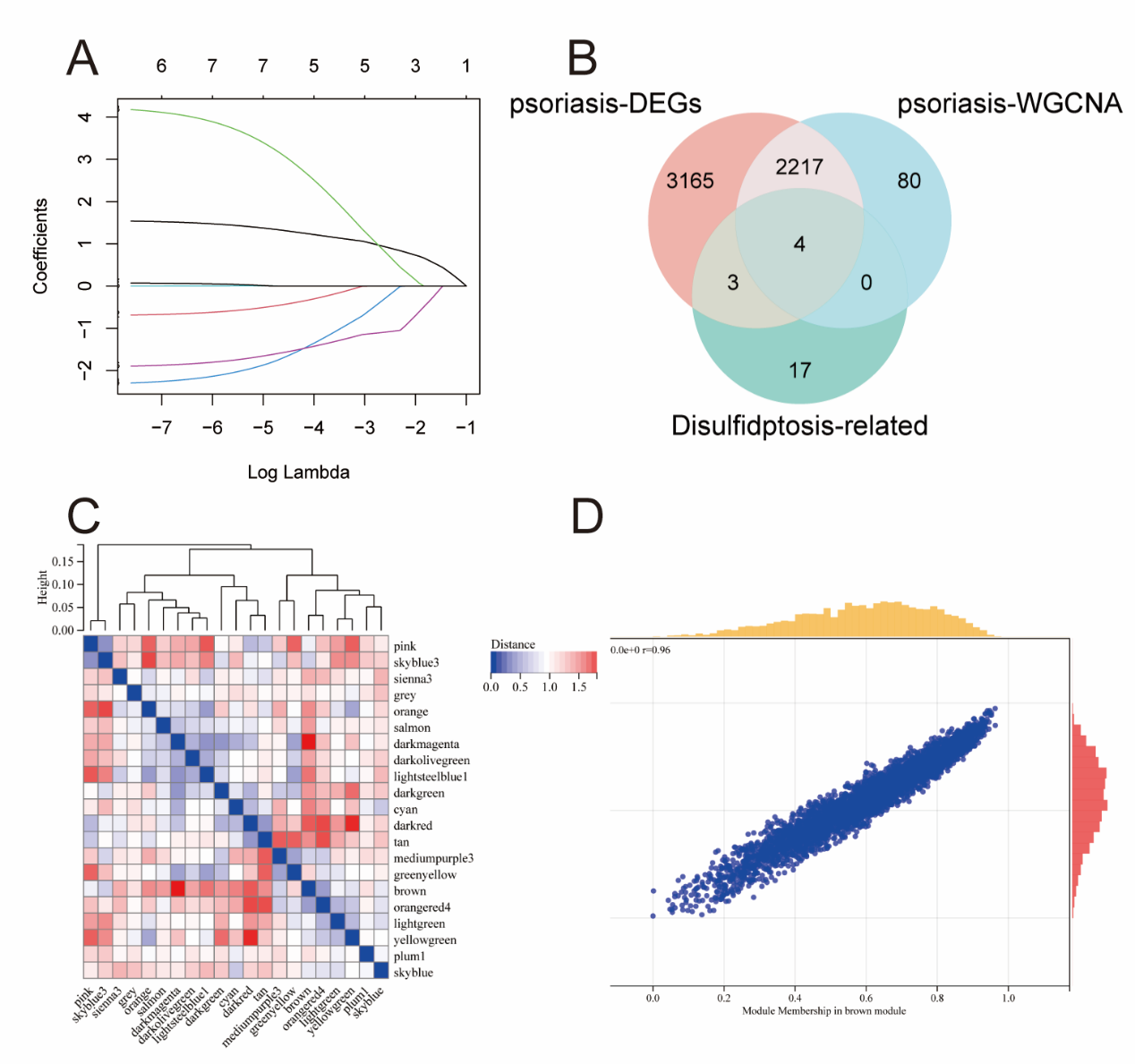
**

**Figure S2. Identification of disulfidptosis-related gene modules in psoriasis.**

(A) LASSO coefficient profiles across log (Lambda) values, showing stable gene selection trajectories.

(B) Venn diagram showing 80 overlapping genes between psoriasis DEGs and WGCNA modules.

(C) Dendrogram and heatmap identifying 15 co-expression modules with color-coded correlations.

(D) Scatter plot showing strong correlation between brown module genes and disulfidptosis-related gene membership.

**
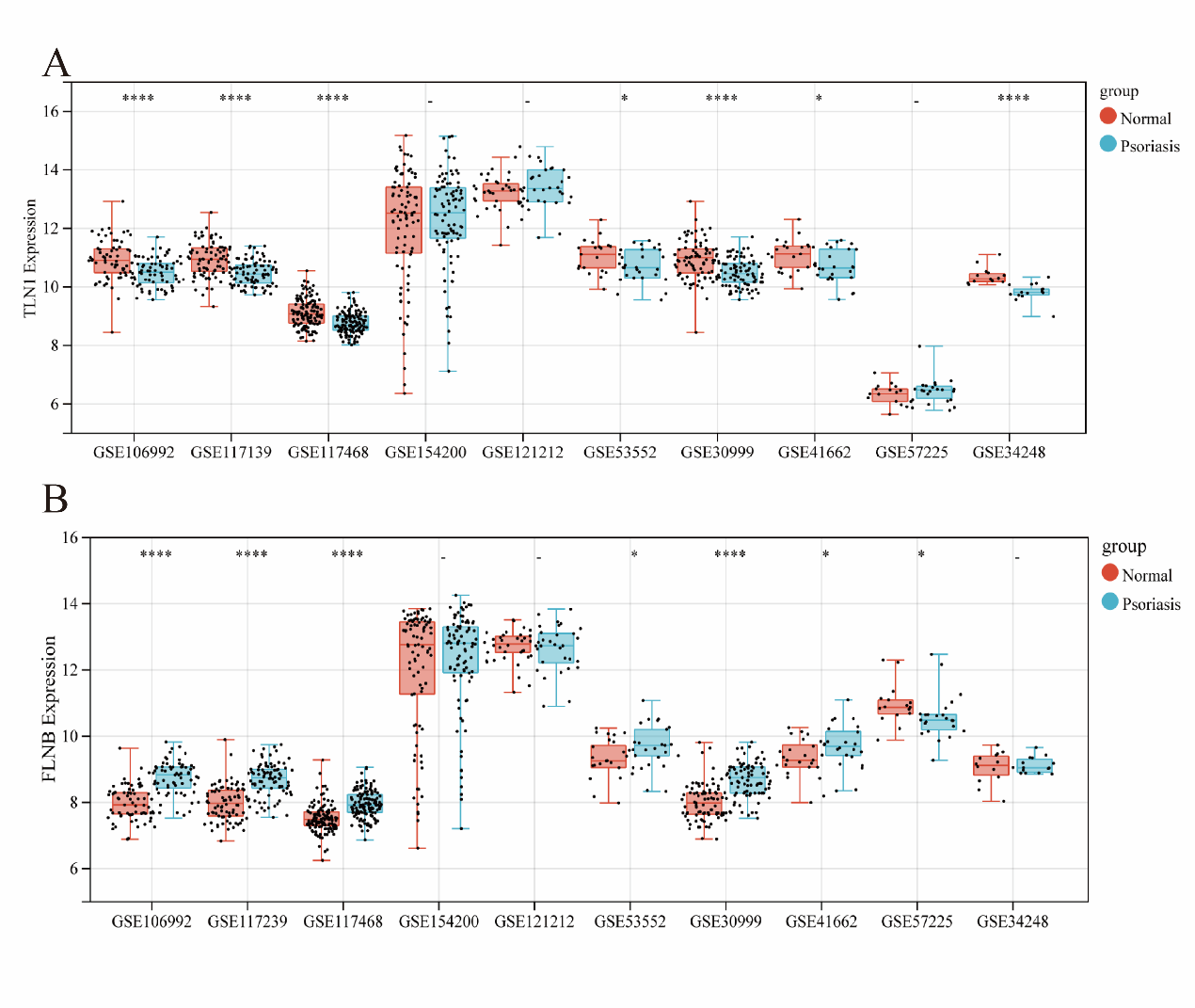
**

**Figure S3. Expression validation of TLN1 and FLNB in psoriasis datasets.**

(A-B) Expression levels of (A) TLN1 and (B) FLNB across multiple GEO datasets.


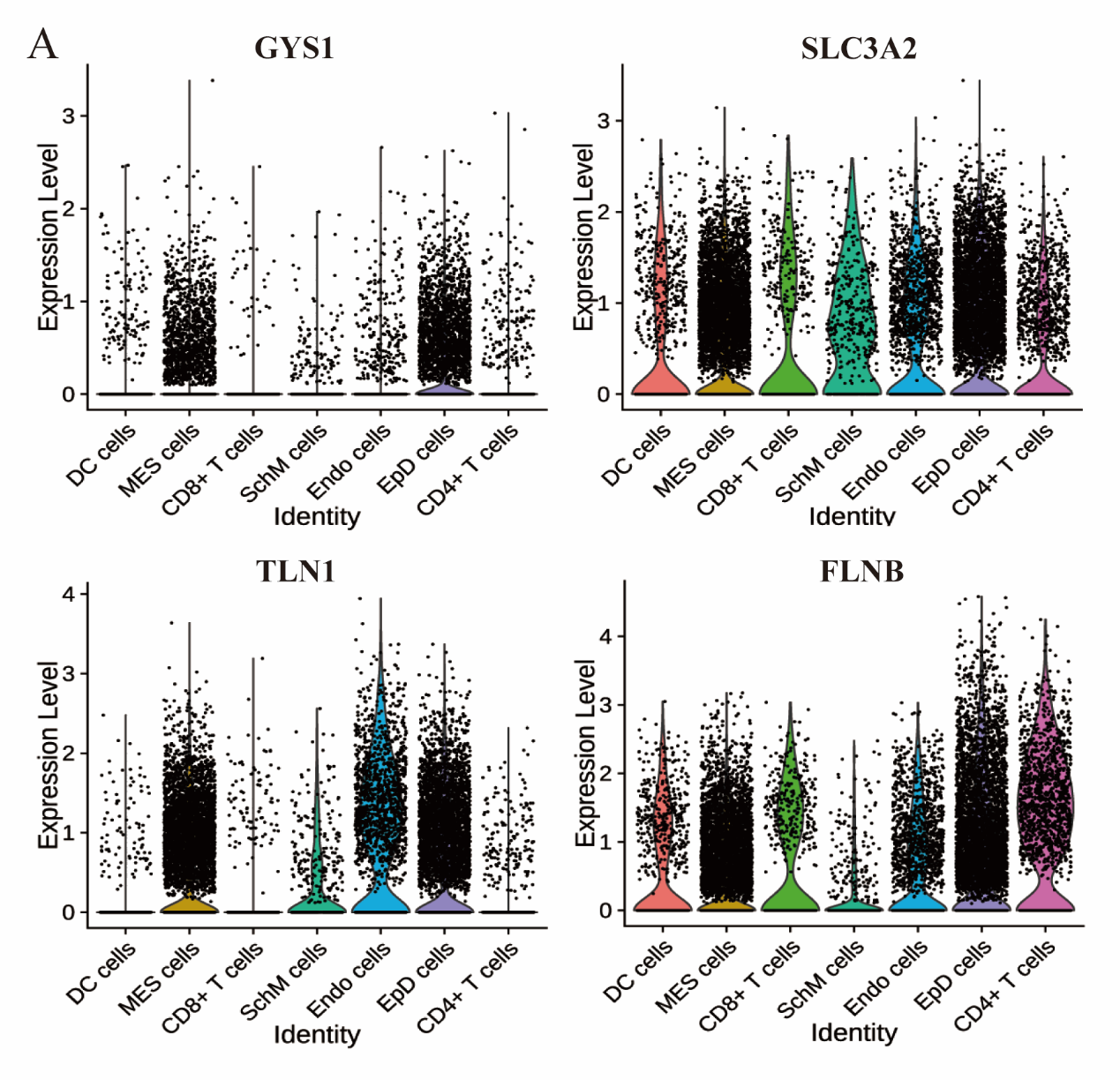


**Figure S4. Disulfidptosis-related hub genes expression in different cell subsets.**

(A-D) Expression levels of (A) GYS1, (B) SLC3A2, (C)TLN1 and (D) FLNB in different cell subsets that identified by UMAP analysis.
