# Supplementary figures and images for: Identification of Disulfidptosis‐Associated Hub Genes in Psoriasis via Integrated Transcriptomic and Experimental Validation Approaches
Source: J Cell Mol Med. 2025 Nov 12;29(21):e70945. doi: 10.1111/jcmm.70945 (PMC12611608; doi:10.1111/jcmm.70945)

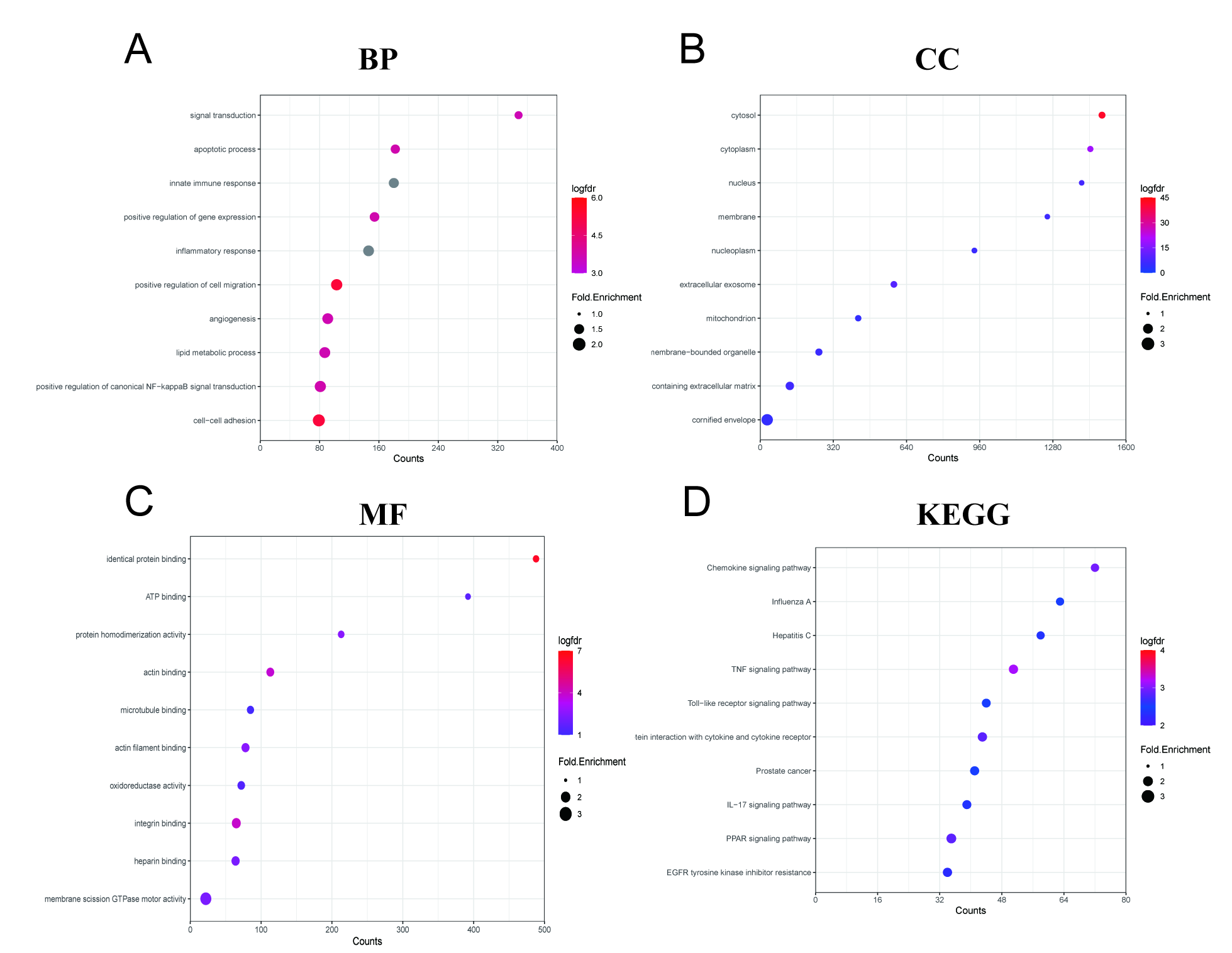

Supplement: Supplementary file 1 — Figure S1: Functional enrichment of DEGs in psoriasis. Figure S2: Identification of disulfidptosis‐related gene modules in psoriasis. Figure S3: Expression validation of TLN1 and FLNB in psoriasis datasets. Figure S4: Disulfidptosis‐related hub genes expression in different cell subsets. [file JCMM-29-e70945-s003.zip › jcmm70945-sup-0001-FigureS1@Supplementary Figure1.tif]

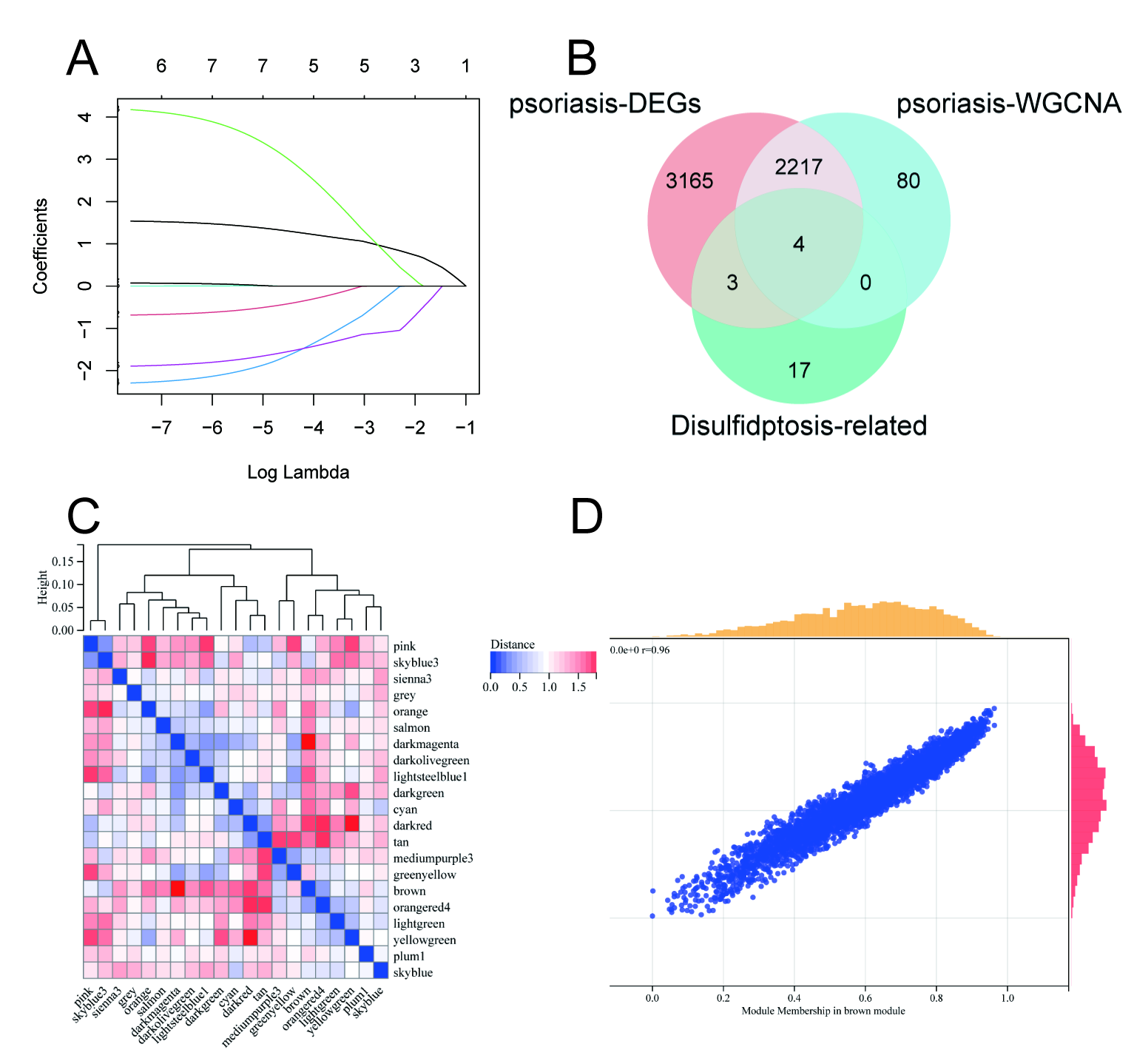

Supplement: Supplementary file 1 — Figure S1: Functional enrichment of DEGs in psoriasis. Figure S2: Identification of disulfidptosis‐related gene modules in psoriasis. Figure S3: Expression validation of TLN1 and FLNB in psoriasis datasets. Figure S4: Disulfidptosis‐related hub genes expression in different cell subsets. [file JCMM-29-e70945-s003.zip › jcmm70945-sup-0002-FigureS2@Supplementary Figure2.tif]

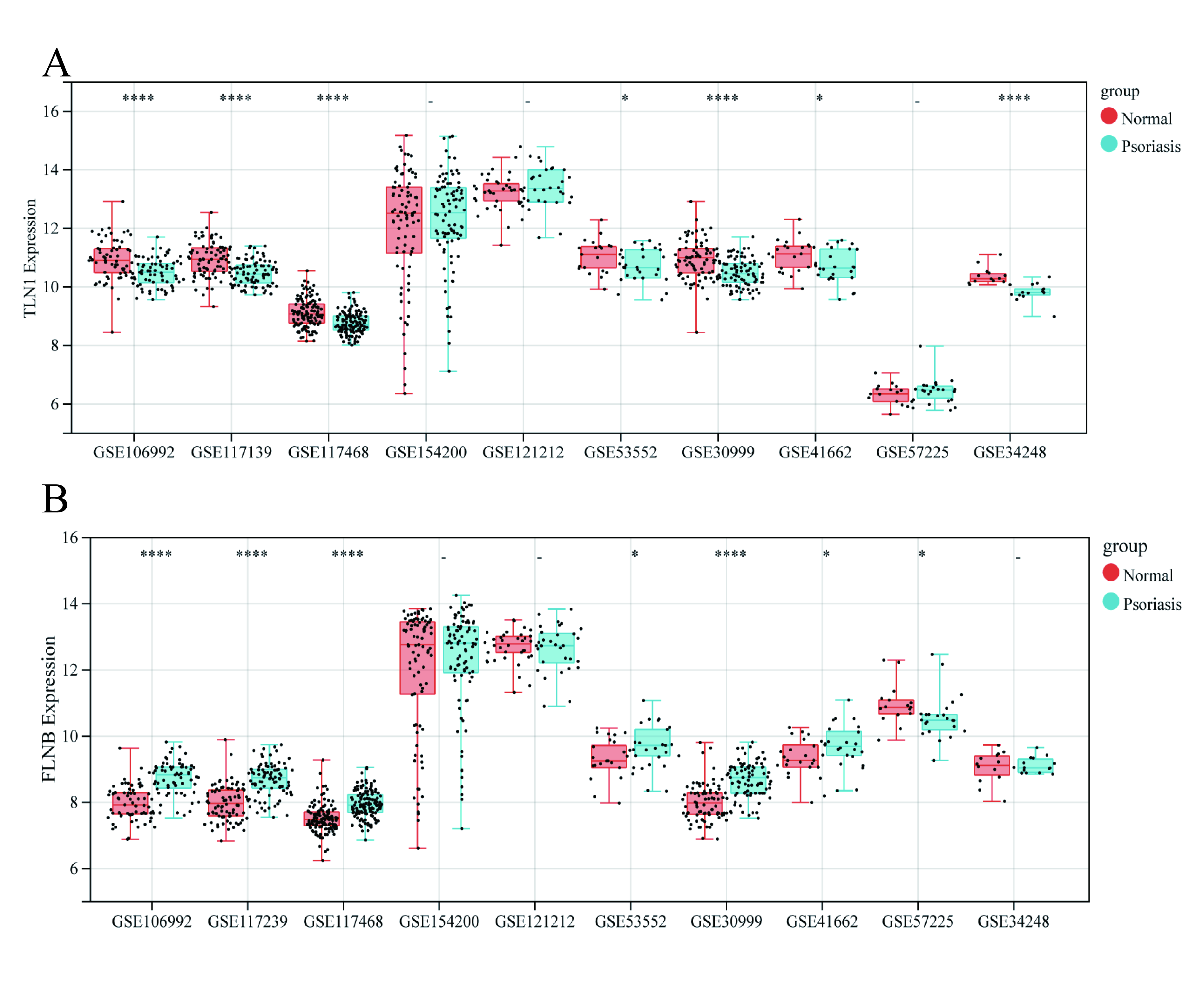

Supplement: Supplementary file 1 — Figure S1: Functional enrichment of DEGs in psoriasis. Figure S2: Identification of disulfidptosis‐related gene modules in psoriasis. Figure S3: Expression validation of TLN1 and FLNB in psoriasis datasets. Figure S4: Disulfidptosis‐related hub genes expression in different cell subsets. [file JCMM-29-e70945-s003.zip › jcmm70945-sup-0003-FigureS3@Supplementary Figure3.tif]

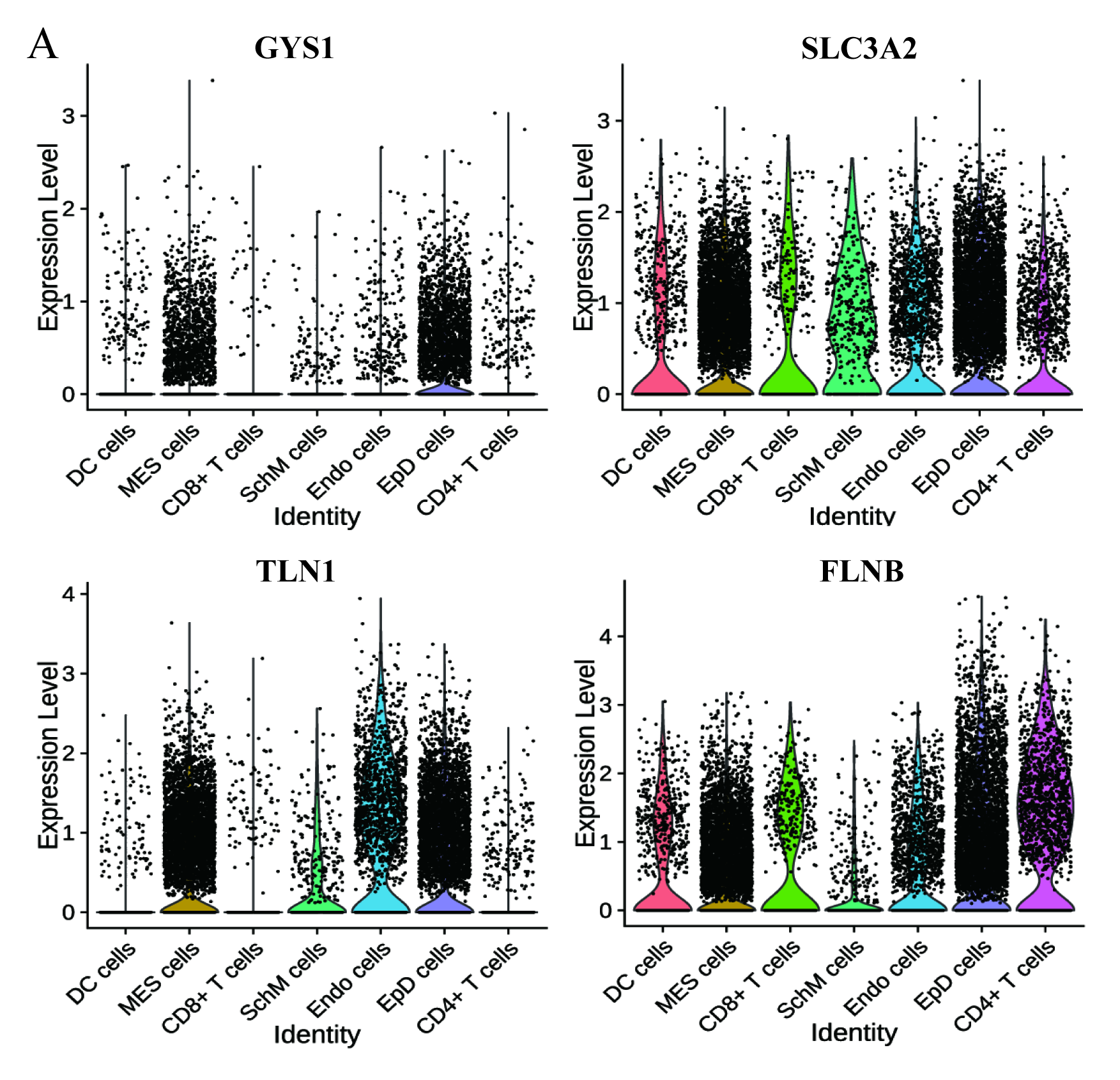

Supplement: Supplementary file 1 — Figure S1: Functional enrichment of DEGs in psoriasis. Figure S2: Identification of disulfidptosis‐related gene modules in psoriasis. Figure S3: Expression validation of TLN1 and FLNB in psoriasis datasets. Figure S4: Disulfidptosis‐related hub genes expression in different cell subsets. [file JCMM-29-e70945-s003.zip › jcmm70945-sup-0004-FigureS4@Supplementary Figure4.tif]
